# Earthworm‐Inspired Soft Skin Crawling Robot

**DOI:** 10.1002/advs.202400012

**Published:** 2024-04-15

**Authors:** Jonathan Tirado, Cao Danh Do, Joséphine Moisson de Vaux, Jonas Jørgensen, Ahmad Rafsanjani

**Affiliations:** ^1^ SDU Soft Robotics, Biorobotics Section The Maersk McKinney Moller Institute University of Southern Denmark Odense 5230 Denmark; ^2^ Department of Mechanics École Centrale de Marseille Marseille 13013 France

**Keywords:** earthworm‐inspired skin, friction anisotropy, limbless locomotion, soft robotics

## Abstract

Earthworms are fascinating animals capable of crawling and burrowing through various terrains using peristaltic motion and the directional friction response of their epidermis. Anisotropic anchoring governed by tiny appendages on their skin called setae is known to enhance the earthworm's locomotion. A multi‐material fabrication technique is employed to produce soft skins with bristles inspired by the earthworm epidermis and their setae. The effect of bristles arranged in triangular and square grids at two spatial densities on the locomotion capability of a simple soft crawling robot comprised of an extending soft actuator covered by the soft skin is investigated experimentally. The results suggest that the presence of bristles results in a rostral to caudal friction ratio of µ_*R*_/µ_*C*_ > 1 with some variations across bristle arrangements and applied elongations. Doubling the number of bristles increases the robot's speed by a factor of 1.78 for the triangular grid while it is less pronounced for the rectangular grid with a small factor of 1.06. Additionally, it is observed that increasing the actuation stroke for the skin with the high‐density triangular grid, from 15% to 30%, elevates the speed from 0.5 to 0.9 mm s^−1^, but further increases in stroke to 45% may compromise the durability of the actuators with less gains in speed (1 mm s^−1^). Finally, it is demonstrated that a crawling robot equipped with soft skin can traverse both a linear and a curved channel.

## Graphical abstract

Earthworm‐inspired modular artificial skins are integrated into a soft linear actuator to investigate the effect of friction anisotropy on limbless locomotion. The skins feature stiff bristles in different arrangements on a soft shell fabricated using multi‐material silicone casting. Comprehensive experiments reveal the role of bristle arrangements and density, stroke, and actuator geometry on the robot's locomotion speed.

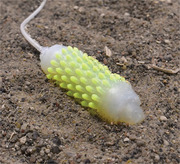

## 1 Introduction

Earthworms move across various terrains by coordinating the contraction of their circular and longitudinal muscles in a motion known as retrograde peristalsis. During locomotion, the earthworm expands segments of its body radially against the burrow walls, to facilitate anchoring by means of tiny bristles on its skin called setae.^[^ ^1^, ^2^, ^3^, ^4^, ^5^, ^6^ ^]^ Two work components—namely, the inertial work required to move the body parts forward and the work needed to overcome ground friction—in combination facilitate the limbless locomotion of earthworms.^[^ ^7^, ^8^ ^]^

Anisotropic friction is ubiquitous in nature, and asymmetric forces play a crucial role in adhesion, locomotion, and transportation.^[^ ^9^, ^10^, ^11^ ^]^ Asymmetric microstructures on surfaces are oriented at specific angles to the supporting layer, creating directional anisotropy in the friction response along the movement direction.^[^ ^12^, ^13^, ^14^ ^]^ Examples of anisotropic friction include the highly ordered microstructures found in various biological systems, such as reptiles,^[^ ^15^, ^16^ ^]^ insects,^[^ ^17^, ^18^, ^19^ ^]^ and worms,^[^ ^20^, ^21^, ^22^, ^23^ ^]^ exhibiting high friction forces for propulsion and low friction forces for sliding. The magnitude of anisotropic friction depends primarily on surface topography, the ratio of sample–substrate stiffness, the aspect ratio of surface structures, and substrate roughness.^[^ ^24^ ^]^ Earthworms generate anisotropic friction through bristles or setae that protrude during the contraction of longitudinal muscles. The interaction between these setae and the substrate enhances anchoring while crawling on the ground or through burrows, facilitating propulsion in a preferred direction.^[^ ^25^, ^26^ ^]^

Earthworms have served as a source of inspiration for various soft robots designed for limbless locomotion.^[^ ^8^, ^27^, ^28^, ^29^ ^]^ Many of these robots aimed to replicate the earthworm's two‐anchor crawling gait, utilizing diverse soft actuation methods. For instance, Calderon et al.^[^ ^30^ ^]^ developed an earthworm‐inspired soft robot featuring two bulging actuators alongside a soft extending actuator reinforced with O‐rings. This design successfully achieved two‐anchor locomotion in horizontal, inclined, and vertical pipes. Das et al.^[^ ^31^ ^]^ created a soft robot using braided artificial muscles, akin to McKibben actuators, capable of generating compression or elongation peristalsis based on the initial braid angle. In another approach, Niiyama et al.^[^ ^32^ ^]^ fabricated an artificial hydrostat composed of a 3D‐printed soft cylindrical chamber filled with an incompressible liquid (water). This system, combined with shape memory alloy (SMA) actuators, mimicked the contraction of circular muscles in earthworms. Additionally, Saga et al.^[^ ^33^ ^]^ developed a peristaltic crawling robot by linking silicone rubber segments longitudinally restrained with Kevlar fibers, specifically designed for in‐pipe crawling. Seok et al.^[^ ^34^ ^]^ created a soft robot by employing SMA coil actuators arranged around a tubular braided mesh to simulate the muscle contraction mechanism observed in earthworms.

Several studies have focused on enhancing anchoring force during locomotion by developing directional anisotropic hook mechanisms. Asawalertsak et al.^[^ ^35^ ^]^ introduced a soft robot featuring an asymmetric abdominal surface structure powered by a single bending actuator and assessed various sawtooth models, substrates, and activation frequencies to achieve adaptable locomotion through narrow spaces. Menciassi et al.^[^ ^36^ ^]^ developed an earthworm‐inspired miniature soft robot composed of SMA‐actuated segments equipped with passive hook‐shaped frictional microstructures. This design allowed the robot to navigate uneven substrates using peristaltic motion. Kim et al.^[^ ^37^ ^]^ presented a microrobot utilizing an SMA spring actuator and a silicone bellow to emulate earthworm muscles through a contraction–extension motion. Moreover, microneedles were used to mimic earthworm setae. Rafsanjani et al.^[^ ^38^ ^]^ created a crawler robot covered by a kirigami skin, enabling anisotropic friction as spike‐like structures emerged during expansion, facilitating directional locomotion. Liu et al.^[^ ^39^ ^]^ constructed a soft earthworm robot by combining a kirigami skin with radially expanding pneumatic actuators, specifically designed for burrowing cohesive soil environments. Additionally, Manwell et al.^[^ ^40^ ^]^ introduced a worm‐like soft robot comprising a steerable mesh driven by tendons and artificial setae structures made from plastic fiber rods, exhibiting locomotion on horizontal and inclined surfaces.

The examples provided above illustrate empirical methods for imparting soft actuators with anisotropic friction to enhance the locomotion of limbless crawling robots. However, there is currently a lack of a systematic quantitative analysis of how the arrangement and distributions of artificial setae on the skin of a soft crawling robot affect its frictional properties and locomotion performance. In this study, we address this gap by developing a soft skin equipped with an array of bristles inspired by earthworms, aiming to mimic both the elasticity of the earthworm epidermis and the rigidity of its setae. The modularity of the skin allows for exclusive and accurate characterization of the effect of artificial setae on friction anisotropy and the locomotion performance of crawling soft robots. We have established a multi‐material fabrication process capable of producing diverse skins with various bristle arrangements and densities. The modular design of the skin allows for easy interchangeability, as each skin can be mounted on top of an extending actuator. Our investigation entails a comprehensive analysis of the friction response exhibited by the skin‐clad actuators at different levels of deformation. Additionally, we compare the observed friction response with locomotion performance and demonstrate crawling in both linear and curved channels (see Video S1, Supporting Information).

## 2 Results and Discussion

### 2.1 Design of the Modular Soft Skins

**Figure 1:**
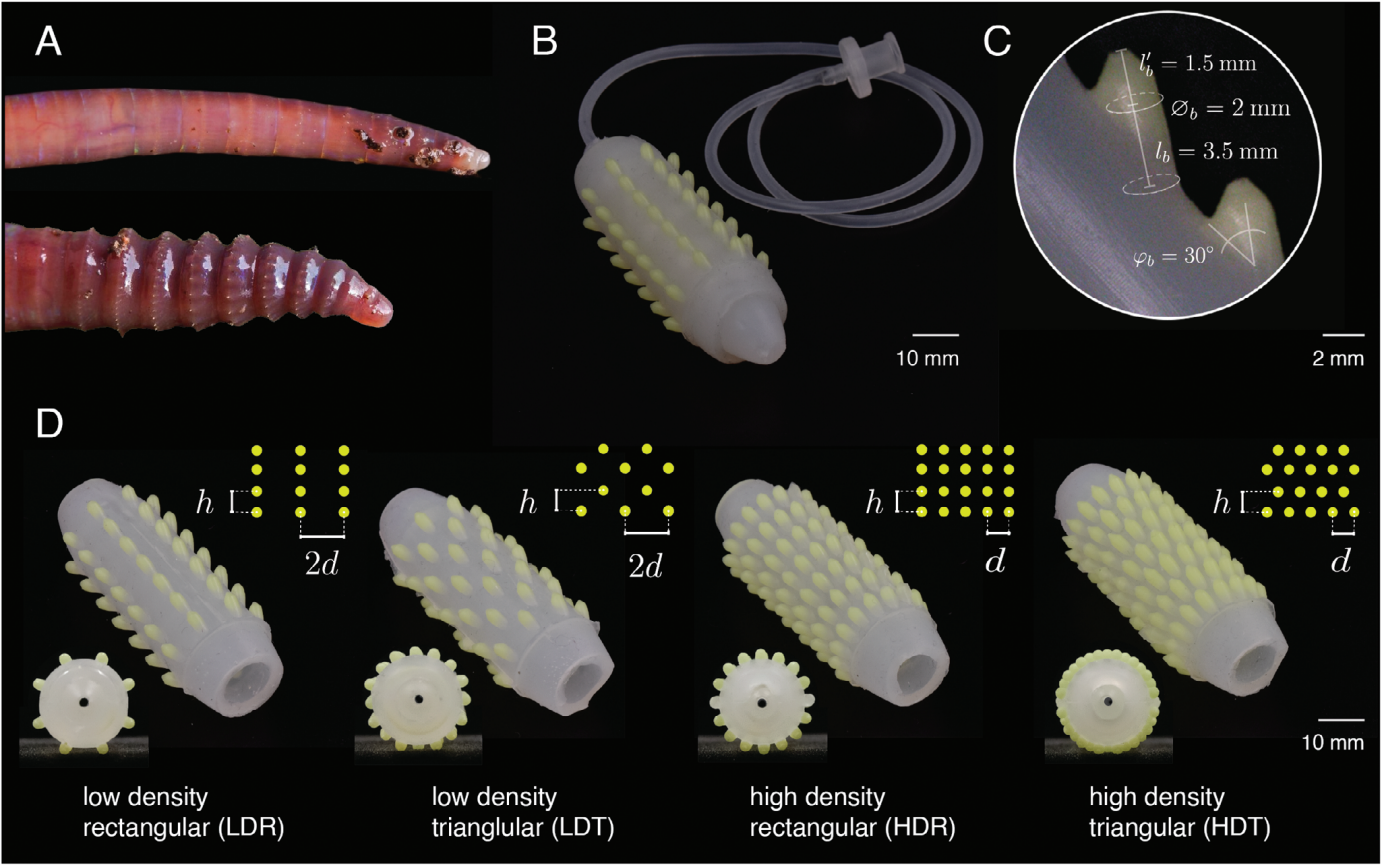
Earthworm‐inspired modular soft skin. A) Earthworms protrude their setae during locomotion (Copyright Steve Shinn Photography). B) Modular soft skin mounted on an extendable soft actuator. C) Artificial setae modeled as an inclined bristle. D) Isometric and cross‐section views of different bristle configurations for soft skins and corresponding geometrical parameters.

The skin design was inspired by the setae of earthworms (**Figure** **1**A) that are known to enhance gripping and assist locomotion through friction modulation.^[^ ^7^, ^25^ ^]^ To test different setae arrangements, we opted for a modular design. This configuration allowed for changing the robot's skin and isolating its effect on frictional properties and locomotion performance while utilizing the same actuator (Figure 1B). We mimicked the setae with an inclined bristle made of a stiffer material than the skin (Figure 1C). We arranged bristles in two configurations based on rectangular and triangular grids at two different densities to investigate both the effect of the pattern and the number of bristles on locomotion behavior (Figure 1D). The geometrical parameters of bristles are the axial spacing *h*, the circumferential spacing *d*, and the inclination angle φ_*b*_. To minimize the number of parameters, we assumed *h*  =  4 mm, *d*   =  2.95 mm, and φ_*b*_ =  30° across all patterns while for low‐density patterns we doubled circumferential spacing to 2*d*. These arrangements resulted in *N_r_*  =  7 circumferential rows of bristles, and *N_l_* =  8, and *N_h_* =  16 axial rows of bristles for low‐ and high‐density patterns, respectively. Throughout this article, we refer to these patterns as high‐density triangular (HDT), low‐density triangular (LDT), high‐density rectangular (HDR), and low‐density rectangular (LDR) patterns (Figure 1D).

### 2.2 Fabrication

**Figure 2:**
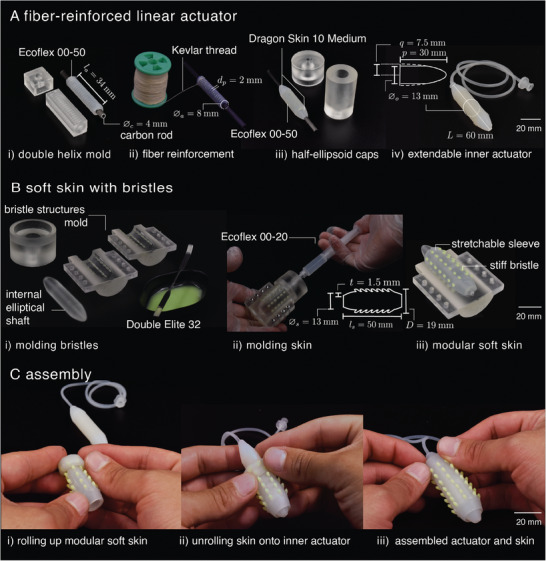
Fabrication. A) Steps for fabrication of the fiber‐reinforced soft extending actuator: (*i*) first silicone molding step for creating the core, (*ii*) double helical fiber winding, (*iii*) second molding step for attaching end caps, and (*iv*) the tube. B) Steps for fabrication of the bioinspired skin: (*i*) brushing stiffer silicone coating into bristle cavities, (*ii*) injecting softer silicone into the assembled mold, (*iii*) extracting the soft skin from the mold. C) Assembling steps for the crawling actuator: (*i*) rolling up the modular soft skin, (*ii*) covering the extendable actuator with the soft skin, (*iii*) fixing the soft skin extremes on the elliptical caps of the inner actuator.

We created a fiber‐reinforced extendable soft actuator and fabricated a modular soft skin that can be mounted and taken off by manual rolling. Both the actuator and skin were fabricated from silicone rubber of different Shore hardness. To create the actuator, we poured a soft Shore 00–50 silicone rubber (Ecoflex 00–50, Smooth‐On) into a 3D‐printed cylindrical mold (*l_a_* =  34 mm, ∅_*a*_ =  8 mm) with a central cavity (∅_*c*_ =  4 mm) that features a double helical groove (*d*_*p*_= 2 mm) on the mold surface for precise winding of the reinforcing thread around the actuator (**Figure** **2**A i). The cylinder core was cured in a convection oven at 60 °C for 20 min with a carbon fiber rod inserted in the mold cavity. After curing, the cylindrical body was reinforced with Kevlar thread (Figure 2A ii). Next, a second mold was used to cast another layer of silicone fixing the reinforcement thread in place and adding elliptical end caps (*p*  =  30 mm, *q*  =  7.5 mm) at both ends (Figure 2A iii). We sequentially fabricated the frontal cap, the body covering, and the posterior cap. For the caps, we used a Shore 10A silicone rubber (Dragon Skin 10 Medium, Smooth‐On), and for the body covering, we again used a Shore 00–50 silicone rubber (Ecoflex 00–50, Smooth‐On) with the same curing process as before. Finally, we de‐molded the composite actuator, removed the carbon rod, and finished the prototype by attaching a flexible silicone tube and sealing the other end (using Dragon Skin) (see Figure 2A iv).

We fabricated the skin using a 3D‐printed mold to create a stretchable sleeve (*l_s_* =  50 mm, ∅_*s*_ =  13 mm, *t*  =  1.5 mm) with stiff bristles (*l*′_*b*_ =  1.5 mm,   *l_b_* =  3.5 mm, ∅_*b*_ =  2 mm, $\varphi_{b}=30^{\circ }$). The mold consisted of four parts, including two semi‐cylindrical parts bearing bristle cavities and truncated elliptical caps, an internal shaft with elliptically rounded ends matching the actuator dimensions, and an enclosing cap for injecting prepolymer silicone rubber (Figure 2B i). We fabricated the skin using two materials: To mimic the stiff setae we used a stiffer Shore 32A silicone rubber (Elite Double 32, Zhermack) and for the stretchable epidermis we used a softer Shore 00–20 silicone rubber (Ecoflex 00–20, Smooth‐On). First, we brushed the interior of the 3D‐printed molds with the stiffer prepolymer silicone, filling all bristle cavities and cleaning the residual material from the internal walls (Figure 2B ii). Then we assembled the mold and injected the softer silicone rubber and cured the skin in a convection oven at 60 °C for 20 min before demolding (Figure 2B iii) (see Note S1 for details, Supporting Information). To mount the skin, we rolled it up, placed its edge on the frontal cap, and unrolled it to cover the soft actuator (Figure 2C).

### 2.3 Mechanical Characterization

**Figure 3:**
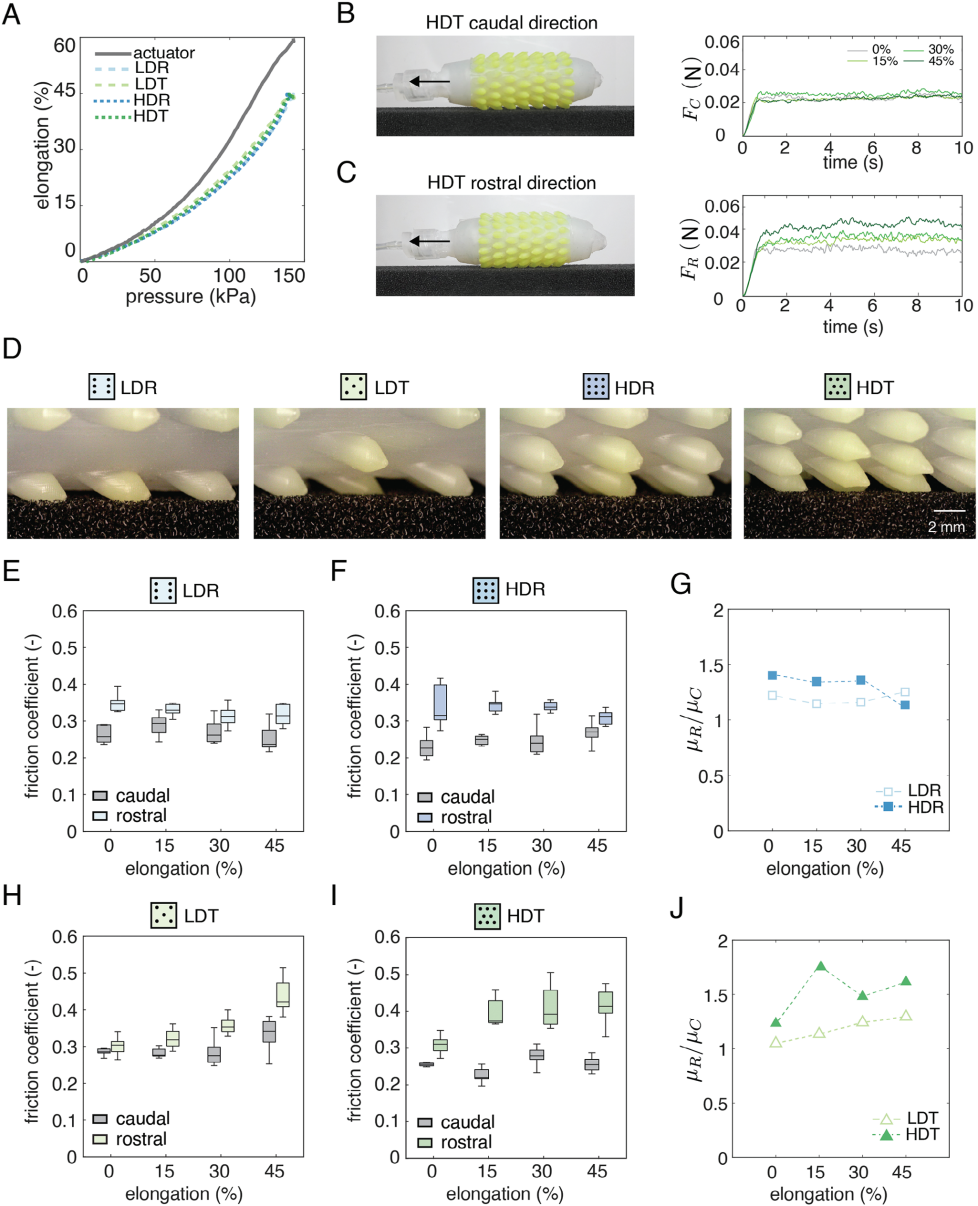
Mechanical characterization. A) Pressure–elongation responses for the actuator without skin and with diverse skin types. B, C) Extendable actuators with HDT skin in caudal (B) and rostral (C) orientations, featuring corresponding friction forces at varying elongation levels. D) Lateral views illustrate different bristle configurations on foam substrate. E, F) Friction coefficients (*n* = 10) at distinct elongation levels in rostral and caudal directions for LDR (E) and HDR (F) skins. G) Directional friction anisotropy ratios for rectangular bristle arrangements (LDR, HDR) across elongation levels based on median values reported in (E) and (F). H, I) Friction coefficients (*n* = 10) at different elongation levels in rostral and caudal directions for LDT (H) and HDT (I) skins. J) Directional friction anisotropy ratios for triangular bristle arrangements (LDT, HDT) at various elongation levels based on median values reported in (H) and(I).

We characterized the pressure–elongation response of the skin‐clad actuator for different skin types to investigate whether the skin has a significant effect on the mechanical properties of the actuator. We used a custom‐built programmable syringe pump (see Note S2 for details, Supporting Information) to inflate the actuator by volumetric injection of 20 mL of air at a constant rate of 2 mL s^−1^. Simultaneously, we recorded the internal pressure using an absolute pressure sensor (MPXH6400A, NXP) and tracked the position of two extremes from videos recorded with a digital camera (D3400, NIKON) using object tracking software in MATLAB (Digital Image Correlation and Tracking ^[^ ^41^ ^]^) (see Note S3 for details, Supporting Information). The elongation percentage, calculated as ε  = 100 × (*l* − *l*_0_)/*l*_0_ , is presented in **Figure** **3**A. Our findings reveal that across all skin types, the entire structure elongates up to 45% of its original length, while the internal pressure reaches 145 kPa. As anticipated, the inclusion of the skin augmented the stiffness and reduced the maximum elongation by ≈15% compared to the bare actuator (gray curve in Figure 3A). These results affirm minimal variation in the mechanical response of actuators across diverse bristle arrangements. Consequently, this suggests the feasibility of employing a common actuation protocol when comparing the performance of extendable actuators covered with different skin types (see Video S2, Supporting Information).

Next, we measured the resistive reaction force of the skin‐clad actuators when pulled by a Kevlar thread (Medium duty 40 Tex, Aramid) using a motorized linear stage (LTS300C, Thorlabs) against a polyurethane foam surface (PPI 60: porosity per inch) when pre‐inflated to different elongations. We measured the friction force with a loadcell (LSB200 miniature high‐performance S‐Beam 5 lbs., FUTEK) mounted on the linear stage while pulling the robot for 100 mm at a constant speed of 10 mm s^−1^ (see Video S3, Supporting Information). We collected 10 datasets for each skin type (LDR, HDR, LDT, HDT) under four different elongation levels (ε  =  0%, 15%, 30%, 45%), in both rostral (against bristles) and caudal (along bristles) directions (see Note S4 for details, Supporting Information). As an illustration, one dataset is visualized in Figure 3B,C, showing the friction forces in caudal (*F_C_*) and rostral (*F_R_*) directions versus time for the HDT pattern at different elongations. Across various elongation levels, compared to the uninflated condition (ε  =  0%), the results indicate a minor variation in the caudal direction ($\Delta F_{C}^{\varepsilon \;=\;15\%}$ = 0.0041 N, $\Delta F_{C}^{\varepsilon \;=\;30\%}$ = 0.0025 N, $\Delta F_{C}^{\varepsilon \;=\;45\%}$ = 0.0001 N), and a more significant difference in the rostral direction $\Delta \;F_{R}^{\varepsilon \;=\;15\%}$= 0.0069 N, $\Delta F_{R}^{\varepsilon \;=\;30\%}$ = 0.009 N, $\Delta \;F_{R}^{\varepsilon \;=\;45\%}$= 0.0112 N.

Figure 3D shows the close‐up views of the actuators with different skin types in contact with a foam surface. In the following, we compare the resulting friction coefficients for each skin type in caudal and rostral directions. Considering that the normal force on a flat surface is proportional to the weight, we determine the friction coefficients by dividing the friction force by the prototype's weight (*W*  =  0.11 N), following the physical relation µ_*C*_ = *F_C_*/*W*  for caudal direction, and µ_*R*_ = *F_R_*/*W*  for rostral direction (see Note S4 for details, Supporting Information). For all skin types and at all elongation levels, we observed that µ_*R*_ > µ_*C*_, indicating the inherent directional friction anisotropy of the skins. For rectangular patterns (Figure 3E,F), we barely observed the effect of elongation on changing friction coefficients, while for triangular patterns (Figure 3H,I), we see an increasing trend in friction coefficients with applied elongation. The anisotropy friction ratio µ_*R*_/µ_*C*_ was generally higher for high‐density patterns than low‐density patterns. The friction anisotropy remained constant for LDR (µ_*R*_/µ_*C*_ ≈ 1.25) and HDR (µ_*R*_/µ_*C*_ ≈ 1.4) configurations (Figure 3G), and gradually increased for LDT (µ_*R*_/µ_*C*_ ≈ 1.1 − 1.3) and HDT (µ_*R*_/µ_*C*_ ≈ 1.4 − 1.6) patterns (Figure 3J) across increasing elongation levels. Among all skin types, the HDT pattern exhibited the highest µ_*R*_/µ_*C*_.

### 2.4 Locomotion in a Linear Channel

**Figure 4:**
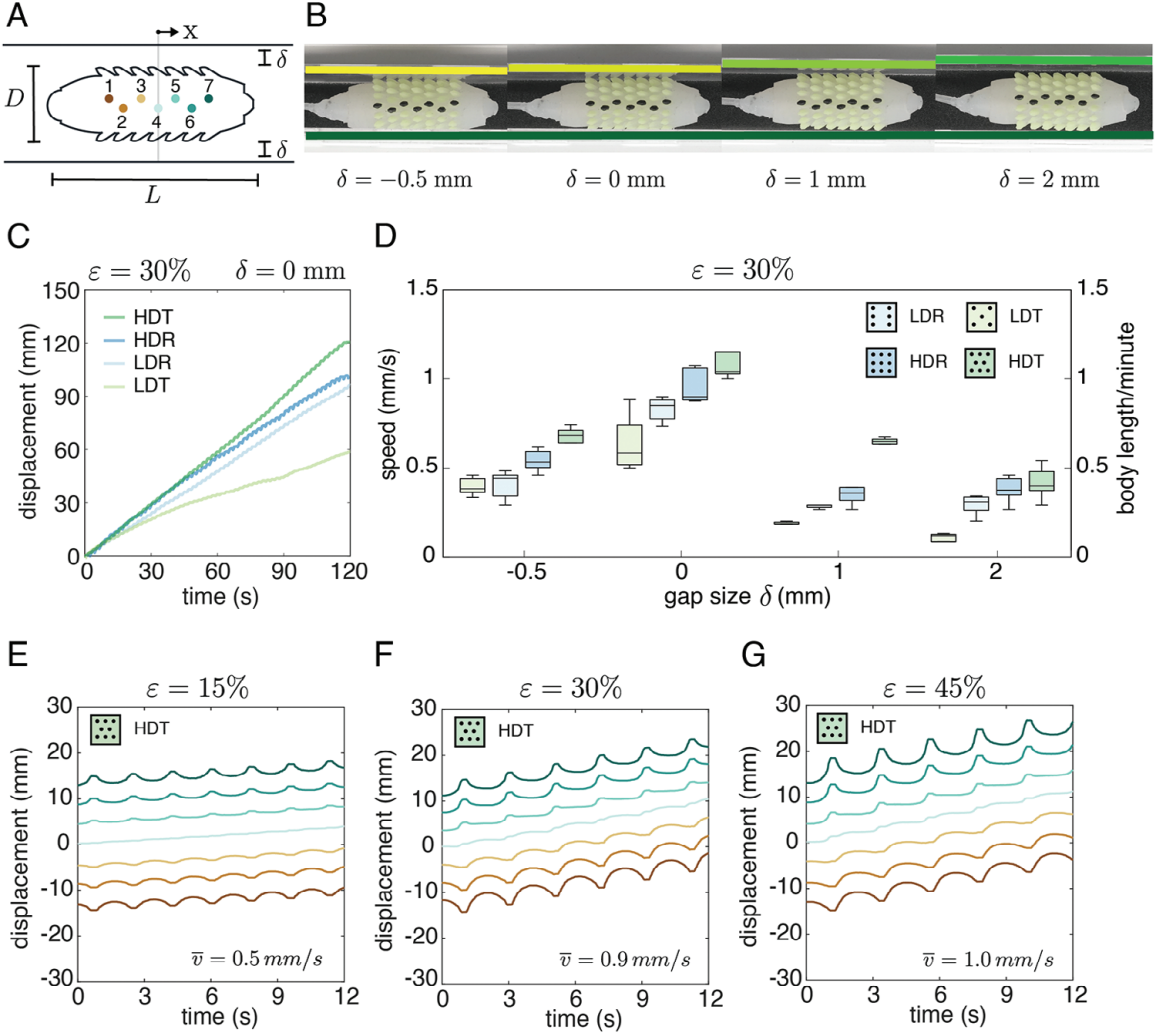
Locomotion in a linear channel. A) Geometrical parameters of the channel and indication of the tracking points for HDT skin, B) Snapshots of the actuator placed in linear channels with different gap sizes. C) Displacement of the middle point of crawling soft robots with different skin types during locomotion in a linear channel with δ  =  0 and an actuation amplitude of ε  =  30%, D) Estimated mean speed (*n* = 5) for all skin types at each gap size δ. E) ε  =  15%, F) ε  =  30%, G) ε  =  45%.

We analyzed the locomotion of the crawling soft robots inside a linear channel constructed from a foam substrate (PPI 60) and two acrylic walls with a prescribed gap, δ, between the tip of the bristles and the walls (refer to **Figure** **4**A). Four gap sizes (δ  =   − 0.5,  0,  1,  2 mm) were considered, as depicted in Figure 4B, and the position of one longitudinal row of bristles (7 points) was tracked with MATLAB (see Note S5 for details, Supporting Information). The soft crawlers were pneumatically actuated using a syringe pump, employing a volume‐controlled protocol that generated a triangular periodic input signal with an amplitude of *A*  =  16 mL and a period of *T*  =  2 s for a duration of 120 s. We calculated the mean velocity (n = 5) of the robots for all skin types at various gap sizes by fitting a line to the displacement of the middle bristle #4 (exemplified for δ  =  0 in Figure 4C) using the least square method approximation. The results shown in Figure 4D indicate that, across all four skin types, the highest speed was attained at δ  =  0. The channel with a negative gap (δ  =   − 0.5 mm) gave the second highest speed and enlarging the gap (δ  =  1 and 2 mm) resulted in slower locomotion. Moreover, higher speeds were observed for skins with denser bristles, with the HDT skin demonstrating the fastest performance ($\bar{v}=\;$0.95 mm s^−1^), followed by the HDR ($\bar{v}=\;$0.89 mm s^−1^), LDT $(\;\bar{v}=\;$0.84) and LDR ($\bar{v}=\;$0.53 mm s^−1^) skins. The results suggest that, for the triangular patterns, doubling the number of bristles increases the robot's speed by a factor of 1.78 while for the rectangular skin, the improvement was small with a factor of 1.06 (see Videos S4 and S5, Supporting Information).

Based on the above results, we employed the HDT pattern to explore the impact of actuation amplitude on locomotion speed. In this investigation, we inflated and deflated the robots using a triangular signal with air volumes corresponding to three elongation levels (ε  =  15%,  30%,  45%). The position of the bristles along the longitudinal axis of the robot was tracked for 12 s. We observed that the robot's speed increased as the elongation level rose, with corresponding mean velocities $\bar{v}=\;$0.5, 0.9, 1 mm s^−1^ for ε  =  15%,  30%,  45%, respectively (see Video S6, Supporting Information). The speed nearly doubled when the elongation amplitude increased from ε  =  15% (Figure 4E) to ε  =  30% (Figure 4E). However, the gain in speed was marginal for further elongation to ε  =  45%, which also posed a risk of damaging the actuator due to excessive pressure (Figure 4G).

**Figure 5:**
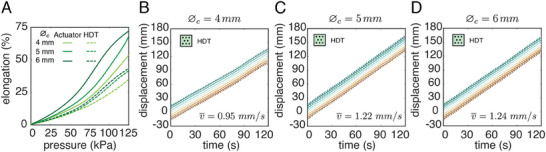
Effect of the geometry of the inner actuator. A) Pressure–elongation curve for actuators with and without HDT skin having different cavity diameters (∅_*c*_ =  4,  5,  6 mm). Corresponding locomotion profiles of crawling robots with HDT skin for B) ∅_*c*_ =  4 mm, C) ∅_*c*_ =  5 mm, and D) ∅_*c*_ =  6 mm.

Next, we investigated the effects of the actuator geometry on the locomotion performance of the robot using the HDT skin for three central cavity diameters ∅_*c*_ =  4,  5,  6 mm. First, we characterized the pressure–elongation curves of these actuators, both with and without HDT skin (see Note S3 for details, Supporting Information). As expected, increasing the size of the cavity results in a larger elongation at a specific pressure and the presence of the skin limits the deformation (**Figure** **5**A). Then, we tracked the robots’ displacement inside a linear channel (δ  =  0) when actuated under a volume‐controlled protocol with a triangular input signal (A  =  16 mL, T  =  2 s) (see Note S5 for details, Supporting Information). The mean velocities were measured as $\bar{v}=\;$0.95, 1.22, 1.24 mm s^−1^ and the maximum elongations were determined as ε  =  30%,  34.5%,  35.3% for ∅_*c*_ =  4,  5,  6 mm, respectively. The results show that the robot's speed was increased by enlarging the central cavity. However, the speed gain from ∅_*c*_ =  4 *mm* to ∅_*c*_ =  5 mm is more pronounced (*28%)* compared to changing from ∅_*c*_ =  5 mm *to* ∅_*c*_ =  6 mm which is merely ≈2% (refer to Figure 5B–D). For the latter diameter, it is likely that the base elastomer material may have reached its limiting strain and thus restricted further extension of the actuator, resulting in only a minor increase in speed (see Video S7, Supporting Information).

### 2.5 Locomotion in a Curved Channel

**Figure 6:**
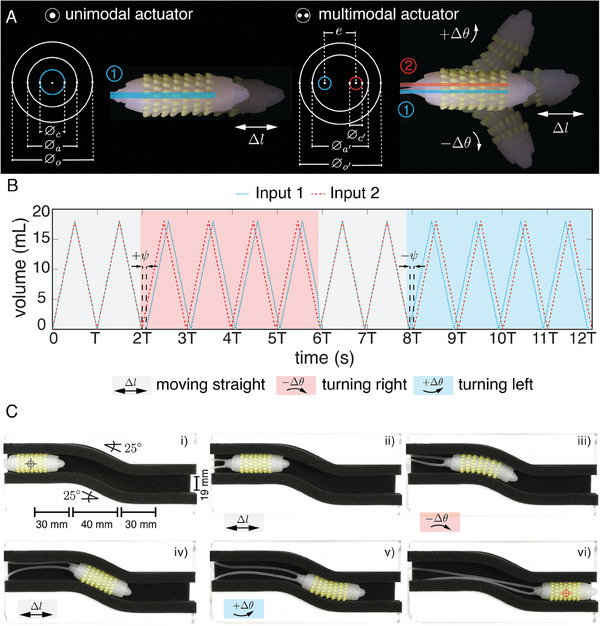
Locomotion in a curved channel, A) Geometrical parameters of the single‐cavity actuator (unimodal) and two‐cavity actuator (multimodal) and their actuation modalities, B) Example of actuation protocol employed to traverse the curved channel (gray: moving straight, red: turning right, blue: turning left), C) Snapshots of the soft crawler at transition points between locomotion modalities. (*i*) start point, (*ii*) Initial linear movement, (*iii*) turning right, (*iv*) intermedial linear movement, (*v*) turning left, (*vi*) endpoint.

After successfully navigating a linear channel, we attempted to traverse a curved channel using the HDT actuator. The considered path comprised three segments with two moderately smooth angles set at φ  = 25° , with no gap between the robot and the walls (δ  =  0). However, our soft crawler failed to complete this task due to its unimodal extending movement, which was unable to adapt to along the path. Consequently, we modified the design by replacing the central cavity with two eccentric chambers placed in parallel with the robot's central axis. This alteration enables differential actuation of the chambers and sideways bending in both directions and also allows synchronous actuation for linear extension like in the previous design. We refer to the original single‐cavity actuator as unimodal and the modified two‐cavity actuator as multimodal (see **Figure** **6**A).

We activated each chamber of the multimodal actuator with a triangular wave having an oscillation period of *T*  =  7 s and an air volume amplitude of *A*  =  18 mL. For the linear part of the path, both chambers were actuated in phase. However, for right and left turning, we introduced a phase shift of ψ  =   − *T*/10 and ψ  =   + *T*/10 for input 2 with respect to input 1, respectively (see Figure 6B). This sequence of actions enabled the multimodal actuator to successfully navigate the entire path and negotiate both inclines along the way in 70 cycles (5 linear, 30 right‐turning, 5 linear, 30 left‐turning) (see Figure 6C; Video S8, Supporting Information).

## 3 Conclusion

In summary, we have presented a bioinspired soft robot design motivated by the setae of earthworms, utilizing a modular soft skin with inclined bristles. The modular design allowed us to explore different bristle arrangements and densities while utilizing a standard fiber‐reinforced soft‐extending actuator. We combined soft and stiff silicone rubbers to fabricate the skin, mimicking the mechanical properties of earthworm epidermis and setae. We characterized the system through pressure–elongation responses, friction force measurements, and locomotion analysis in linear channels with different widths. Friction force measurements showed directional friction anisotropy across all skin types, with higher ratios for high‐density patterns. The locomotion analysis in linear channels demonstrated the impact of skin type and gap size on robot speed, with denser bristle arrangements and a channel without gap resulting in higher speeds. Moreover, we explored the effect of actuation amplitude on locomotion speed, observing an increase in speed with higher elongation levels, although the speed gain was insignificant at extreme elongation levels. We encountered challenges with the unimodal extending movement in the attempt to traverse a curved channel. However, a design modification, i.e., the implementation of two eccentric chambers in the multimodal actuator, enabled successful navigation of a curved path, demonstrating incline adaptability. Overall, our results showcase the versatility of the modular soft skin design, highlighting the importance of bristle patterns and arrangements in influencing frictional properties and locomotion behavior in confined spaces for various applications such as soil sensing, sewer pipe inspection, and minimally invasive biomedical devices.

## Conflict of Interest

The authors declare no conflict of interest.

## Supporting information

Supporting Information

Supplemental Movie 1

Supplemental Movie 2

Supplemental Movie 3

Supplemental Movie 4

Supplemental Movie 5

Supplemental Movie 6

Supplemental Movie 7

Supplemental Movie 8

## Data Availability

The data that support the findings of this study are available in the supplementary material of this article.
