# Supplementary material for: Earthworm‐Inspired Soft Skin Crawling Robot
Source: Adv Sci (Weinh). 2024 Apr 15;11(23):2400012. doi: 10.1002/advs.202400012 (PMC11187925; doi:10.1002/advs.202400012)
Supplement: Supplementary file 1 — Supporting Information [file ADVS-11-2400012-s008.pdf]

ADVANCED SCIENCE
Open Access

Supporting Information

for *Adv. Sci.*, DOI 10.1002/advs.202400012

Earthworm-Inspired Soft Skin Crawling Robot

*Jonathan Tirado, Cao Danh Do, Joséphine Moisson de Vaux, Jonas Jørgensen and Ahmad Rafsanjani**

Supplementary Information

Earthworm-Inspired Soft Skin Crawling Robot

Jonathan Tirado[1], Cao Danh Do[1], Joséphine Moisson de Vaux[1,2], Jonas Jørgensen[1], Ahmad Rafsanjani[1],*

[1] SDU Soft Robotics, Biorobotics Section, The Maersk McKinney Moller Institute, University of Southern Denmark, Odense 5230, Denmark

[2] Department of Mechanics, École Centrale de Marseille, 13013 Marseille, France

* Corresponding author ahra@sdu.dk
Date: March 9, 2024

This document contains:

- Note S1. Mold design
  - Figure S1. Technical drawings of the molds
- Note S2. Programmable syringe pump
  - Figure S2. Custom-built programmable syringe pump
- Note S3. Pressure and elongation data acquisition
  - Figure S3. Pressure – elongation characterization setup
  - Figure S4. Pressure – elongation characterization data
- Note S4. Friction analysis
  - Figure S5. Friction characterization setup
  - Figure S6. Anisotropic friction force analysis
- Note S5. Locomotion analysis
  - Figure S7. Experimental platforms for locomotion analysis
  - Figure S8. Locomotion performance analysis in linear channel
- Note S6. Description of supporting videos

Note S1. Mold design

Figure S1 shows the drawing of the molds used for fabrication of the soft actuator and the skin.

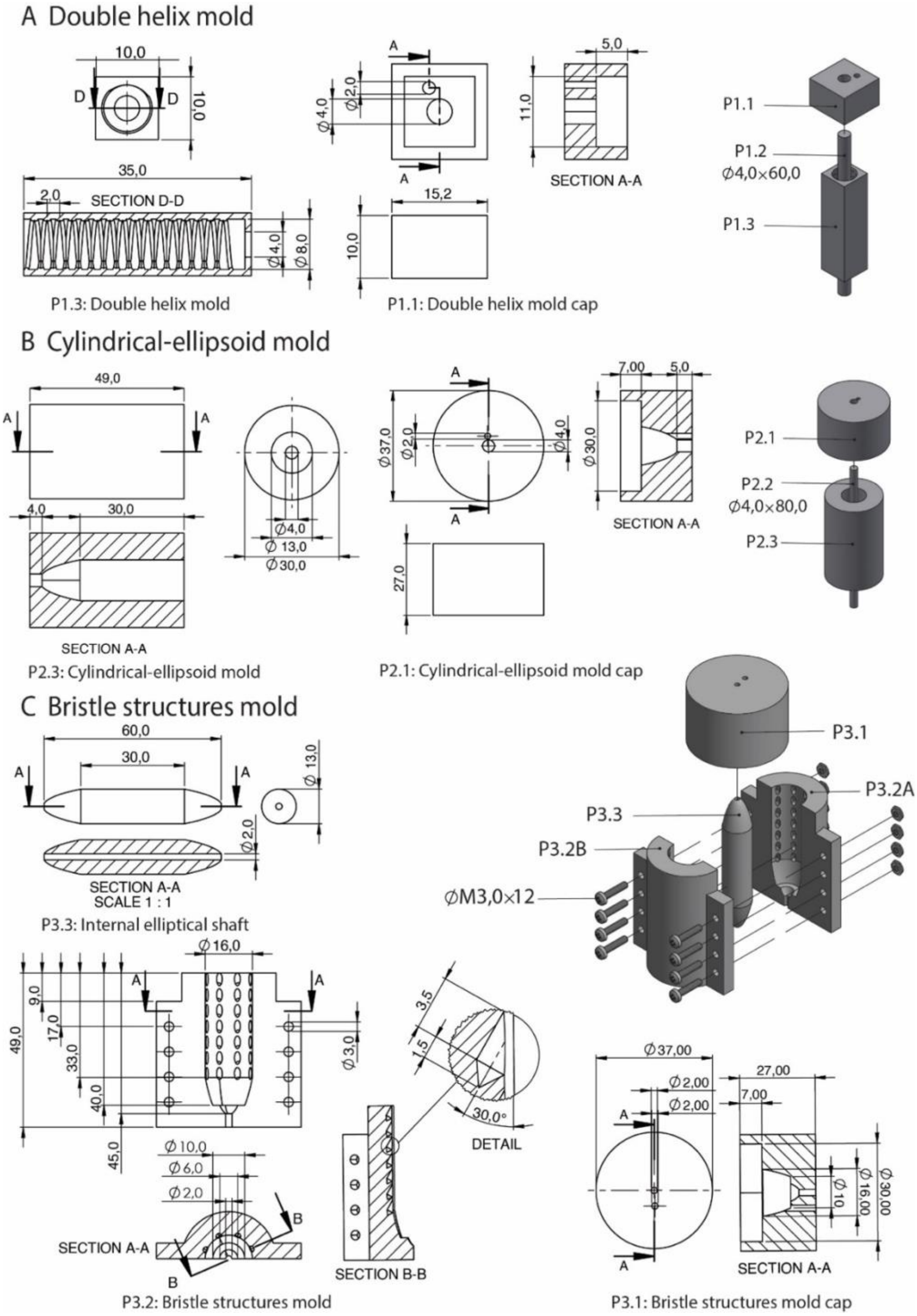

Figure S1. Technical drawings of the molds. **A**. Soft actuator mold with double helix groves, **B**. Cylindrical skin mold with truncated ellipsoidal caps, **C**. Molds for fabrication of bristles over the skin.

Note S2. Programmable syringe pump

We developed a programmable modular syringe pump, designed to facilitate precise fluid dispensing. The system's architecture is based on a motorized linear slide mechanism controlled by a driver board (Bigtreetech Octopus v1.1 3D printer board, 8 stepper driver outputs). The linear stage assembly features 3D-printed holders tailored for plastic syringes (Henke Sass Wolf 50 ml). These syringes are affixed to a linear guide rail carriage (MGN ST15C, length=250 mm), propelled via a rotational lead screw (ACME 8 mm diameter, length=250 mm). The entire linear slide system is housed within an aluminum profile structure (80 mm×20 mm×500 mm) and connected to a stepper motor (NEMA 17) through a flexible coupler (5×8 mm diameter). Power is supplied to the modular syringe pump via a DC power source (24V, 2.5A). The volumetric injection control is achieved through the integration of the control board with a computer system by serial communication. In our application, we employed MATLAB as the primary software interface to generate precise air volume injection sequences simultaneously for 1 or 2 syringes. The MATLAB script computes the necessary linear displacement of the piston syringe to correspond with the specified air volume requirement. Subsequently, it translates these displacement positions into G-code commands, facilitating transmission to the control board through serial communication protocols.

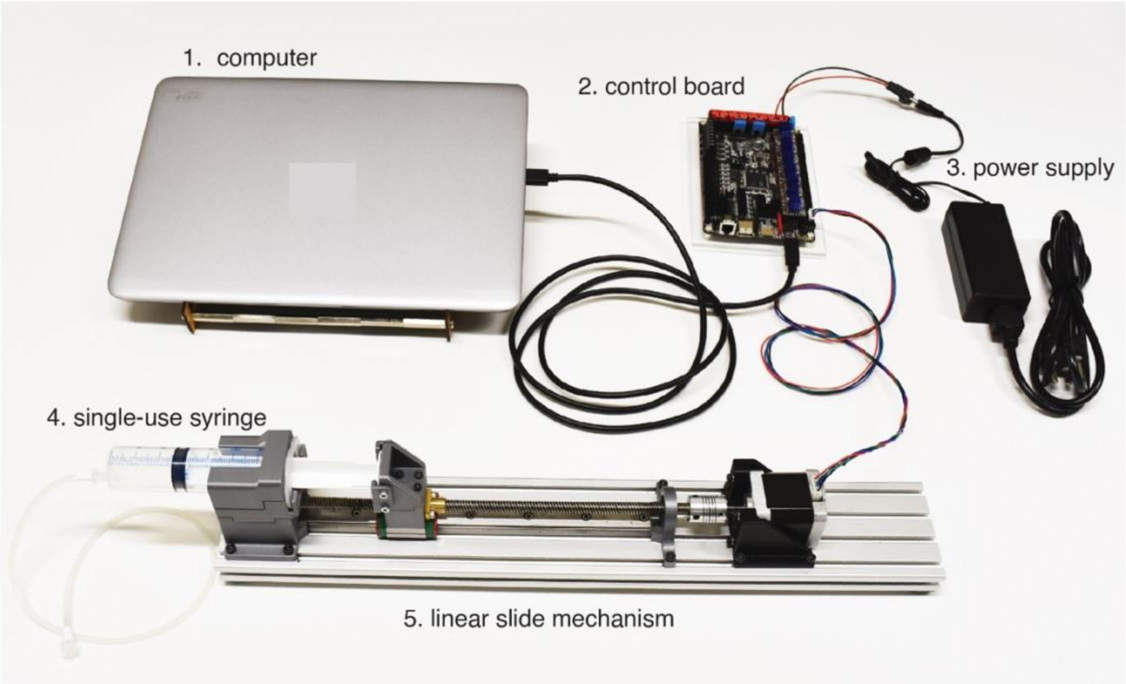

Figure S2. Custom-built programmable syringe pump. Components and electrical connections

Note S3. Pressure and elongation measurements

The actuator pressure and elongations were measured simultaneously, mounting the actuator in a holder structure. For pressure measurements, an absolute pressure sensor (MPXH6400A, NXP) was used, interfaced with a data acquisition card (National Instruments DAQ NI USB-6008). This process was performed using a MATLAB script programmed to log measurements at a sampling rate of 100 samples per second. Elongation analysis involved the initial placement of two markers at the upper and lower extremes of the extendable actuator (see Fig. S3). The actuator's behavior was recorded both in its bare state (without skin) and with various skin types mounted during the extension process. Subsequently, video recordings were processed using Digital Image Correlation and Tracking software [1] in MATLAB environment. This process allowed for the precise identification of the positions of both markers (upper and lower extremes of the actuator). Elongation percentages were calculated based on the original length of the actuator and the distance between the two marker points. This calculation provided insights into the elongation characteristics over time. The resulting datasets depicting the pressure and elongation measurements are visually represented in Fig. S4.A-E. Finally, we eliminated time between two datasets and generated elongation-pressure curve shown in Fig. S4F.

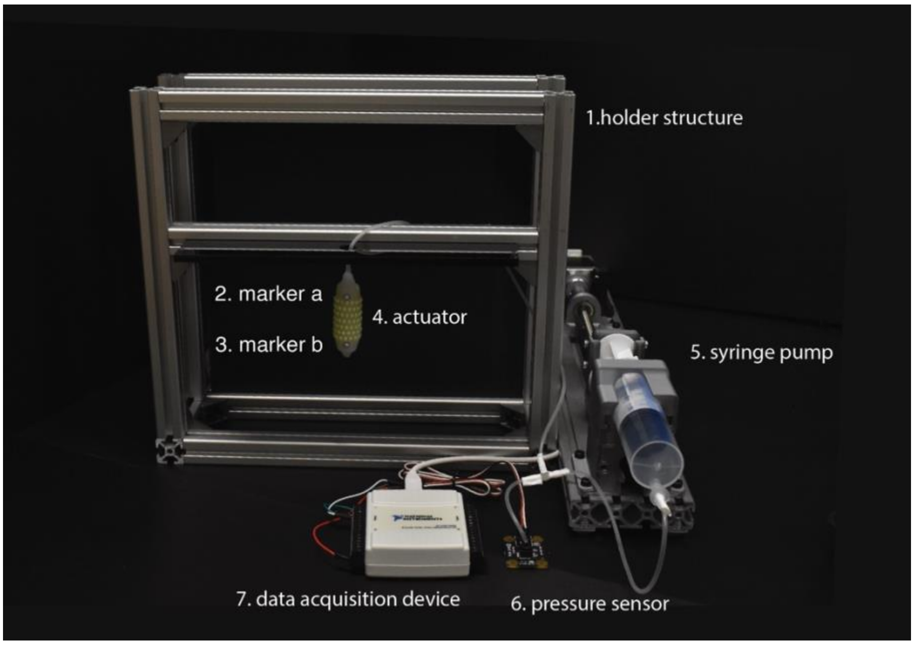

Figure S3. Pressure – elongation characterization setup. Components, mechanic arrangement, pneumatic connections and measurement instruments.

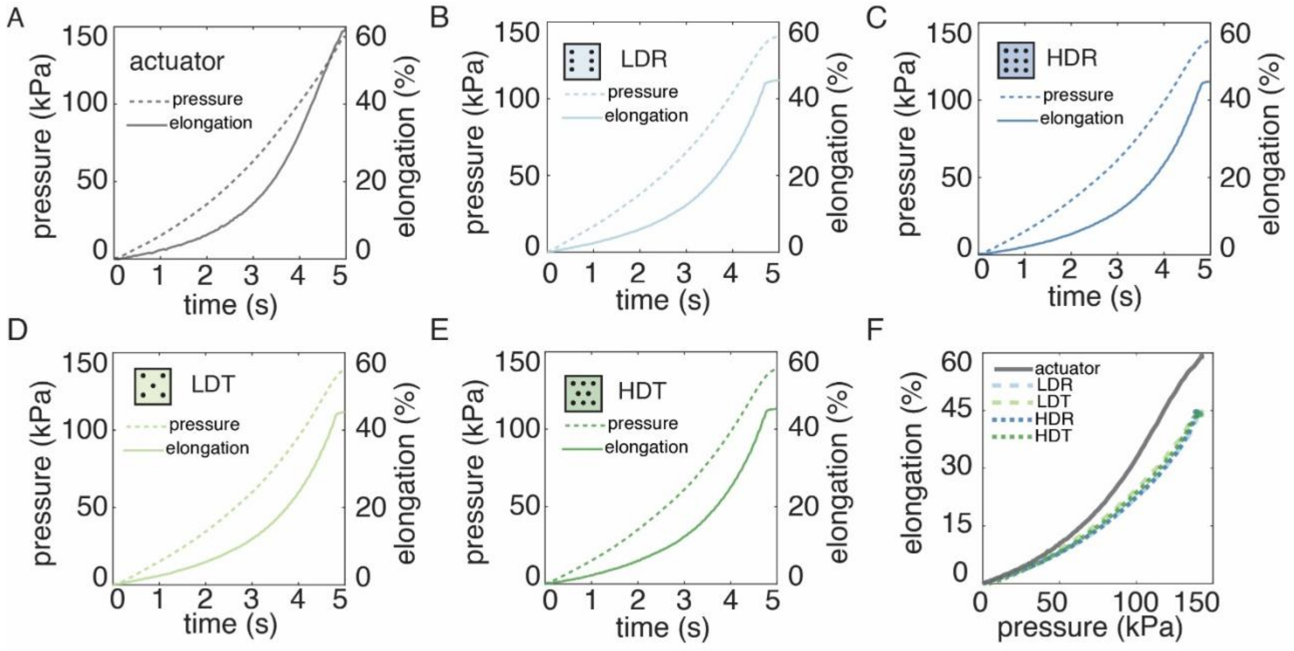

Figure S4. Pressure–elongation measurements. A. Pressure response (dotted line) and elongation response (solid line) for actuator without skin, **B-E.** Pressure and elongation response for LDR (B), HDR (C), LDT (D), HDT (E) skins, **F.** Pressure-elongation responses for the actuator without skin and with different skin types.

Note S4. Friction analysis

To measure the resistive reaction force of the skin-clad actuators against polyurethane foam surfaces (PPI 60: porosity per inch), we built an experimental platform. This setup consists of an acrylic support that provides the foundation for mounting our experimental foam surface, serving as the guided substrate against which the skin-clad actuators interface during the experimental trials. Accompanying this, a motorized linear stage (LTS300C, Thorlabs), paired with an amplified load cell, enables measurement of the friction force while the robot, tethered to the system, undergoes pulling movements. To facilitate controlled inflation of the actuators a syringe pump is employed. The setup needs intricate connections: The robot interfaces with both the loadcell (LSB200 miniature high-performance S-Beam 5 lbs., FUTEK) and the pneumatic output of the syringe pump through silicone pipes. The tubes are reinforced with Kevlar thread within their cavities to mitigate elastic elongation and ensure correct force transmission. Additionally, we use a data acquisition device (National Instruments DAQ NI USB-6008) to read the force response measured with the loadcell. A MATLAB script data logs the friction force and controls the displacement of the motorized linear stage, and the air-volume injection of the syringe pump (see Fig. S5). For this study, we collected 10 datasets for each skin type (LDR, HDR, LDT, HDT) under four different elongation levels ($\varepsilon = 0\%$, 15%, 30%, 45%), in both rostral (against bristles) and caudal (along bristles) directions. All datasets were postprocessed by averaging the 10 most dominant maxima values of each signal that represent the static friction forces. After computing the average value across all datasets, we determined the corresponding friction coefficients from the measured forces. These results were systematically organized by sliding direction and skin type to facilitate statistical representation. We employed the `findpeaks` function in MATLAB (see Fig. S6) and subsequently utilized these findings for statistical analysis.

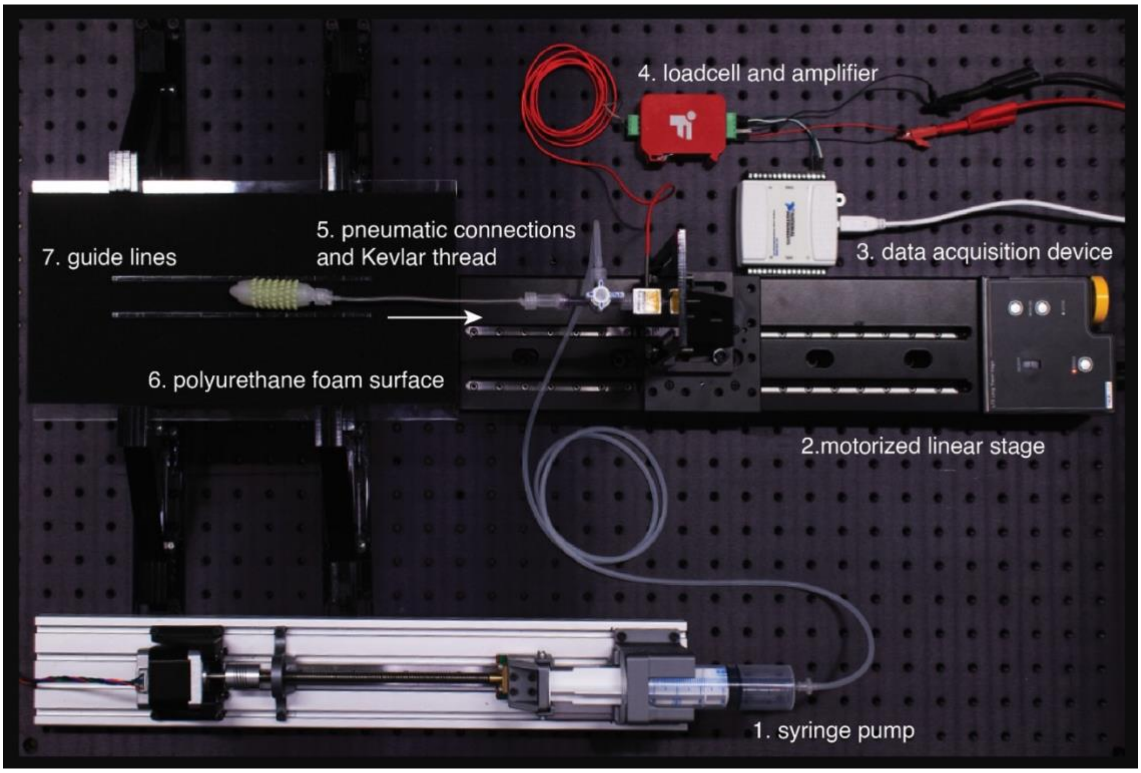

Figure S5. Friction characterization setup. Components, mechanical and pneumatic connections and measurement instruments.

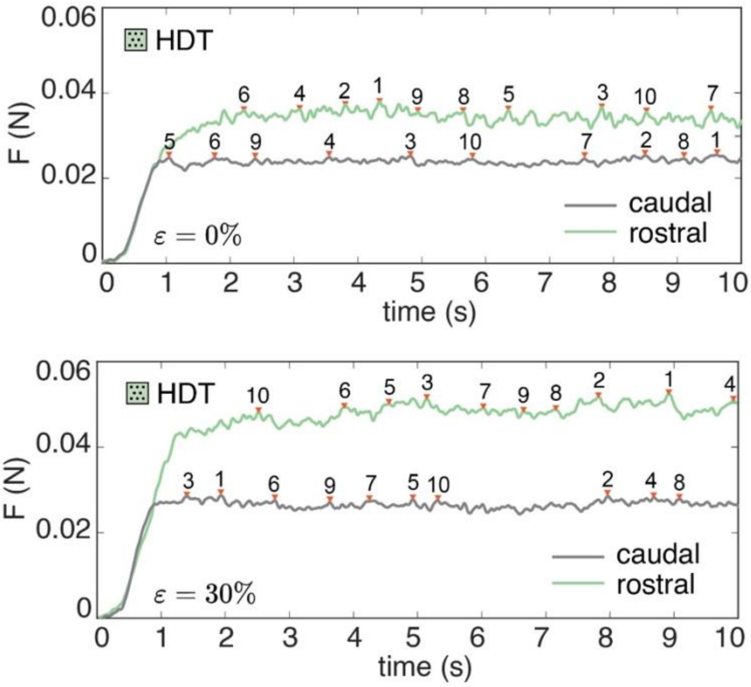

Figure S6. Anisotropic friction force analysis. Extendable actuator with HDT skin in caudal and rostral orientations, featuring 10 highest peaks in friction forces at **A**. $\varepsilon = 0\%$, and **B**. $\varepsilon = 30\%$.

Note S5. Locomotion analysis

To evaluate the locomotion performance of our robotic prototype, we constructed two distinct experimental setups (linear and curved channels) using laser-cut acrylic plates and polyurethane foam surfaces.

Initially, our assessment involved the use of the linear channel setup, where the base featured the same foam surface used for friction characterization (PPI60) while the lateral walls consisted of acrylic plates (see Fig. S7A). For the first stage of the locomotion analysis, we varied the gap size between the lateral walls (δ) (see Fig. 4B) to explore its impact on the crawling speed for each skin type. Then, we extended our study to also analyze the effect of actuation amplitude for the HDT skin type. To estimate the speed of the prototypes, we marked the position of a longitudinal row of bristles (7 points) before recording each of the experimental sequences. Subsequently, utilizing MATLAB's Digital Image Correlation and Tracking software (see Fig. S8A), we tracked their displacement within the linear channel. We fitted a linear curve to the tracking data of the middle bristle (designated as the 4th bristle) using the least squares method and used its slope for speed approximation (see Fig. S8B).

Our second demonstration involved traversing a curved channel using the HDT skin type. This required a redesign of the extendable unimodal actuator, which involved replacing the central cavity with two eccentric chambers aligned parallel to the actuator's central axis, resulting in a multimodal actuator capable of differential actuation. The curved channel setup was constructed using acrylic plates to form the external structure. Both the internal walls of the path and the base of the channel were covered with a foam surface (see Fig. S7B).

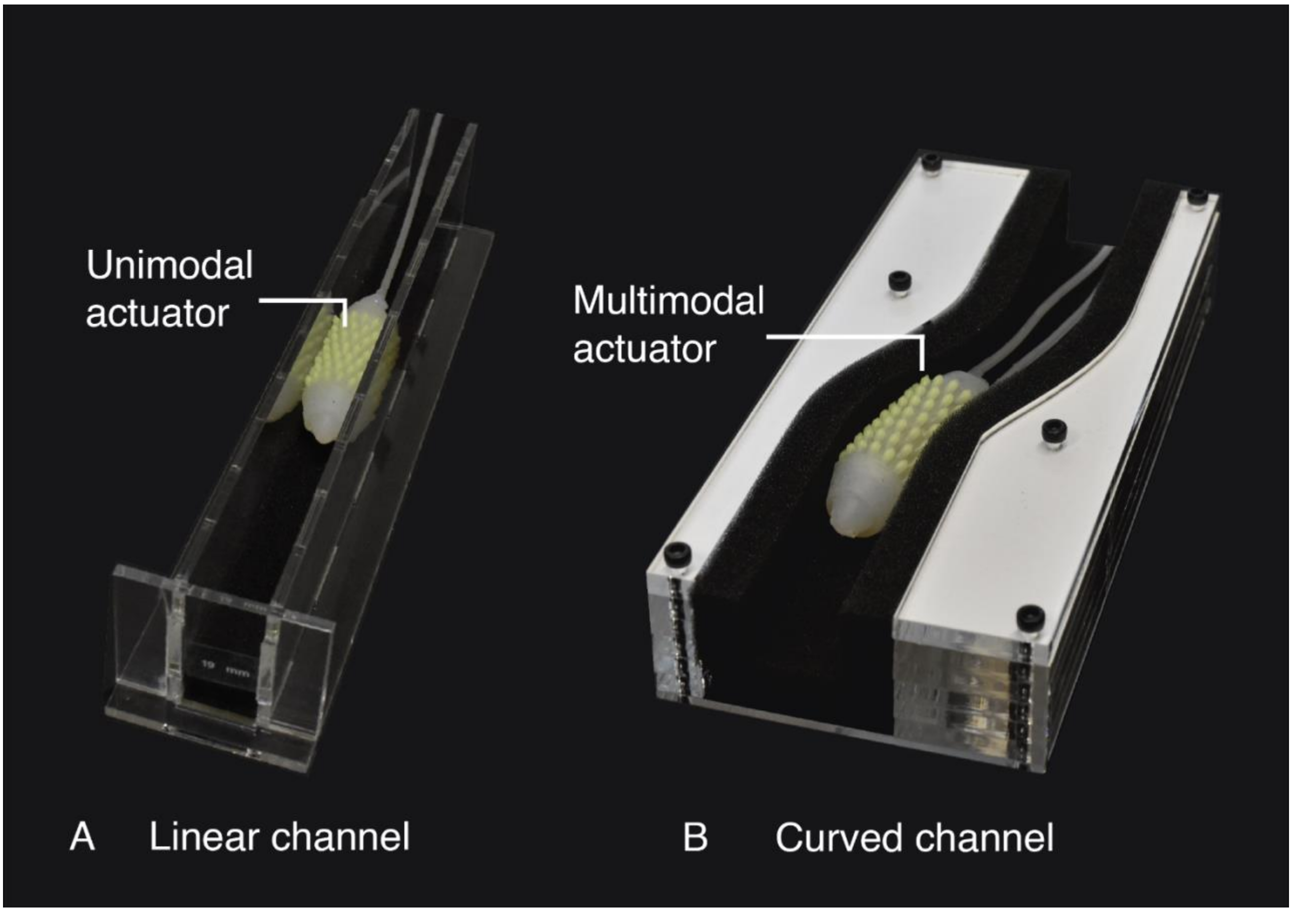

Figure S7. Experimental platforms for locomotion analysis. A. Linear channel and unimodal actuator, **B.** Curved channel, and multimodal actuator.

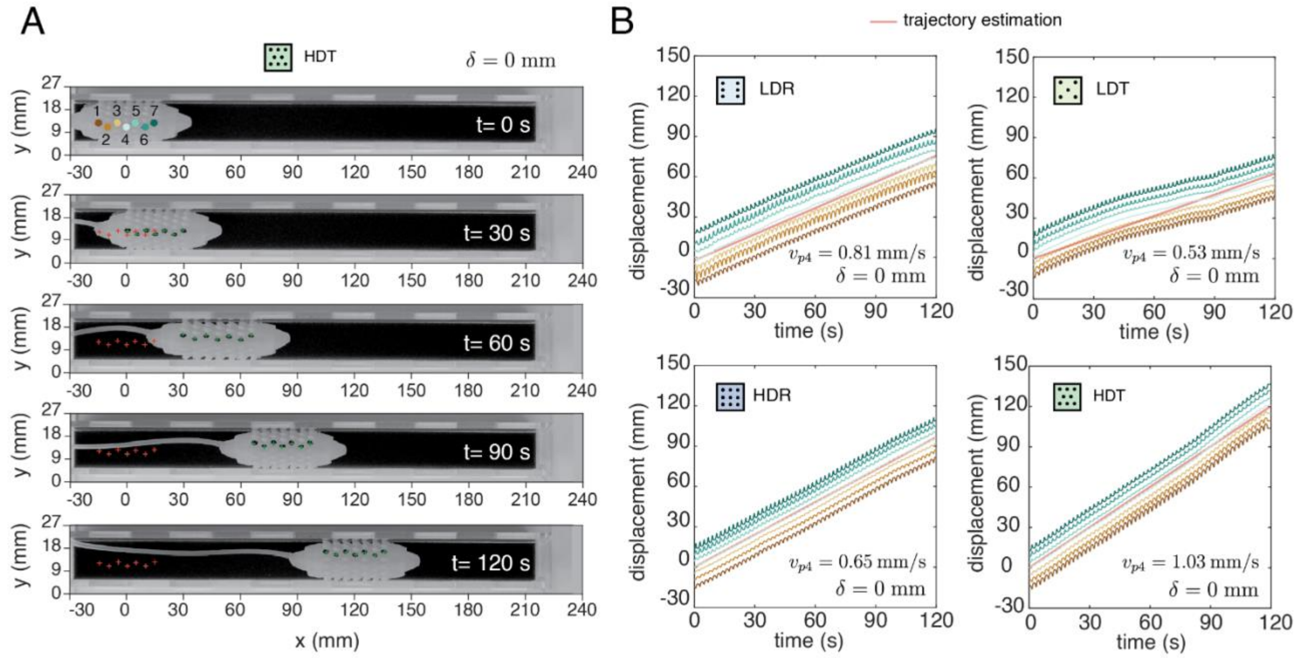

Figure S8. Locomotion performance analysis in linear channel. A. HDT skin type linear channel locomotion for $\delta = 0$. **B**. Displacement of the tracked points for all skin types during locomotion with an actuation amplitude of $\varepsilon = 30\%$, Estimated mean velocity for middle bristle (4th bristle) and fitted trajectory line (red line).

Note S6. Description of supporting videos

Video S1. Bioinspired soft skin demonstration. Actuation of an extendable soft actuator without and with the bioinspired soft skin.

Video S2. Pressure elongation characteristics. Pressure and elongation response for the actuator without and with all the skin types.

Video S3. Friction Analysis, HDT Skin. Friction force response in caudal and rostral direction for the HDT skin robot for $\varepsilon = 0\%$ and $\varepsilon = 30\%$.

Video S4. Linear channel, Skin evaluation. Locomotion analysis for all skin types in a linear channel with the gap size $\delta = 0$ mm at $\varepsilon = 30\%$.

Video S5. Linear channel, HDT skin gap evaluation. Locomotion analysis for the HDT skin robot in four linear channels with different gap sizes ($\delta = -0.5, \delta = 0, \delta = 1, \delta = 2$ mm) at $\varepsilon = 30\%$.

Video S6. Linear channel, Actuation amplitude evaluation. Locomotion analysis for the HDT skin robot in a linear channel for three levels of elongation ($\varepsilon = 15\%, \varepsilon = 30\%$ and $\varepsilon = 45\%$).

Video S7. Effect of the geometry of the inner actuator. Pressure and elongation response for all the geometries of the inner actuator ($\emptyset_c = 4, 5, 6$ mm) without and with the HDT skin. Locomotion analysis for the actuators with HDT skin in a linear channel with the gap size $\delta = 0$ mm under a volume-controlled protocol with a triangular input signal (A = 16 mL, T = 2 s).

Video S8. Locomotion in a curved channel. Locomotion through a curved channel for a multimodal actuator with HDT skin.

References

[1] Christoph Eberl, "Digital Image Correlation and Tracking." MATLAB, Nov. 13, 2023. [MATLAB]. Available: https://se.mathworks.com/matlabcentral/fileexchange/12413-digital-image-correlation-and-tracking
